# Supplementary material for: Targeting PIKfyve-driven lipid homeostasis as a metabolic vulnerability in pancreatic cancer
Source: bioRxiv. 2024 Mar 20:2024.03.18.585580. Preprint. [Version 1] doi: 10.1101/2024.03.18.585580 (PMC10983929; doi:10.1101/2024.03.18.585580)

Extended Data Fig. 1

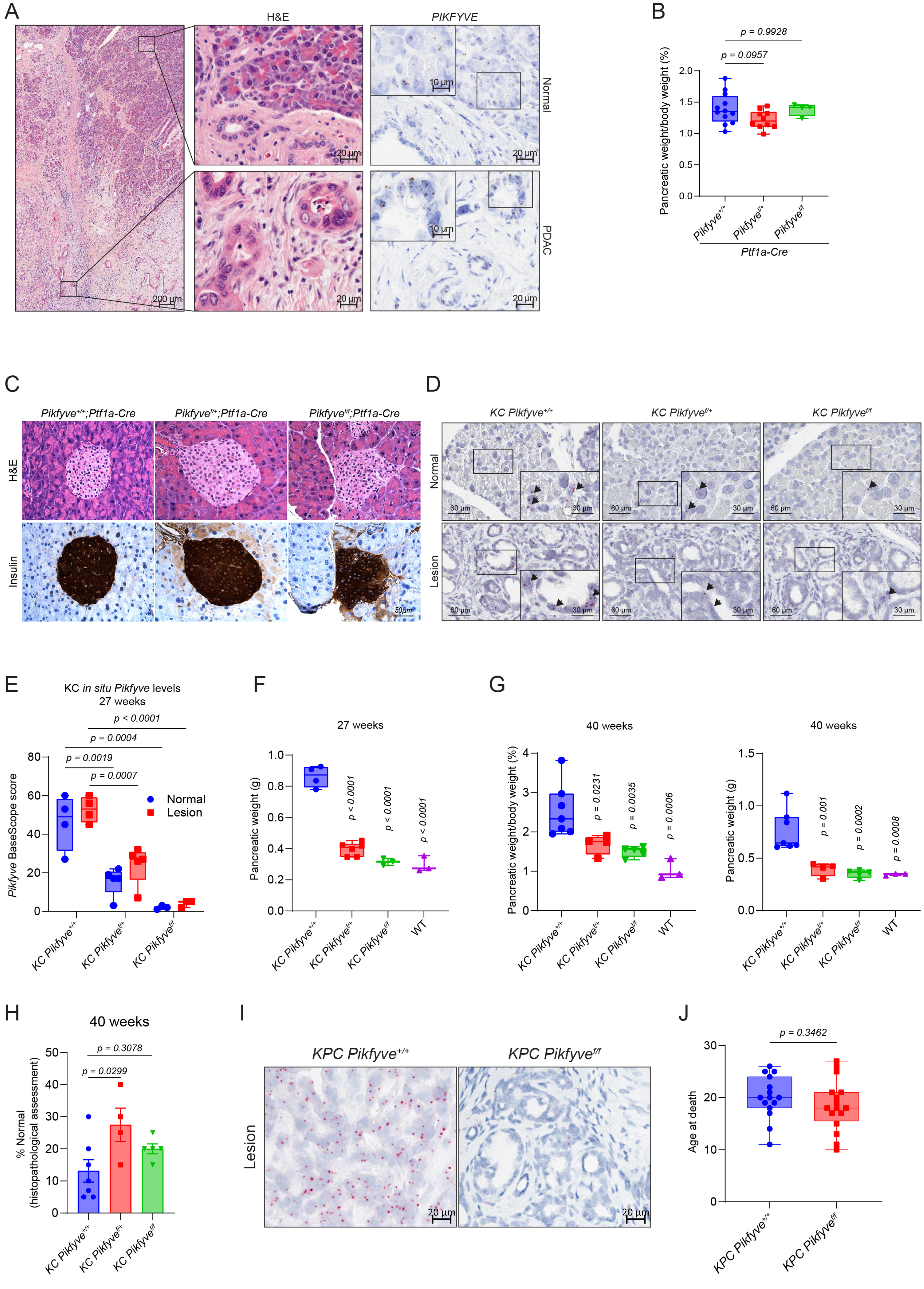

Extended Data Fig. 2

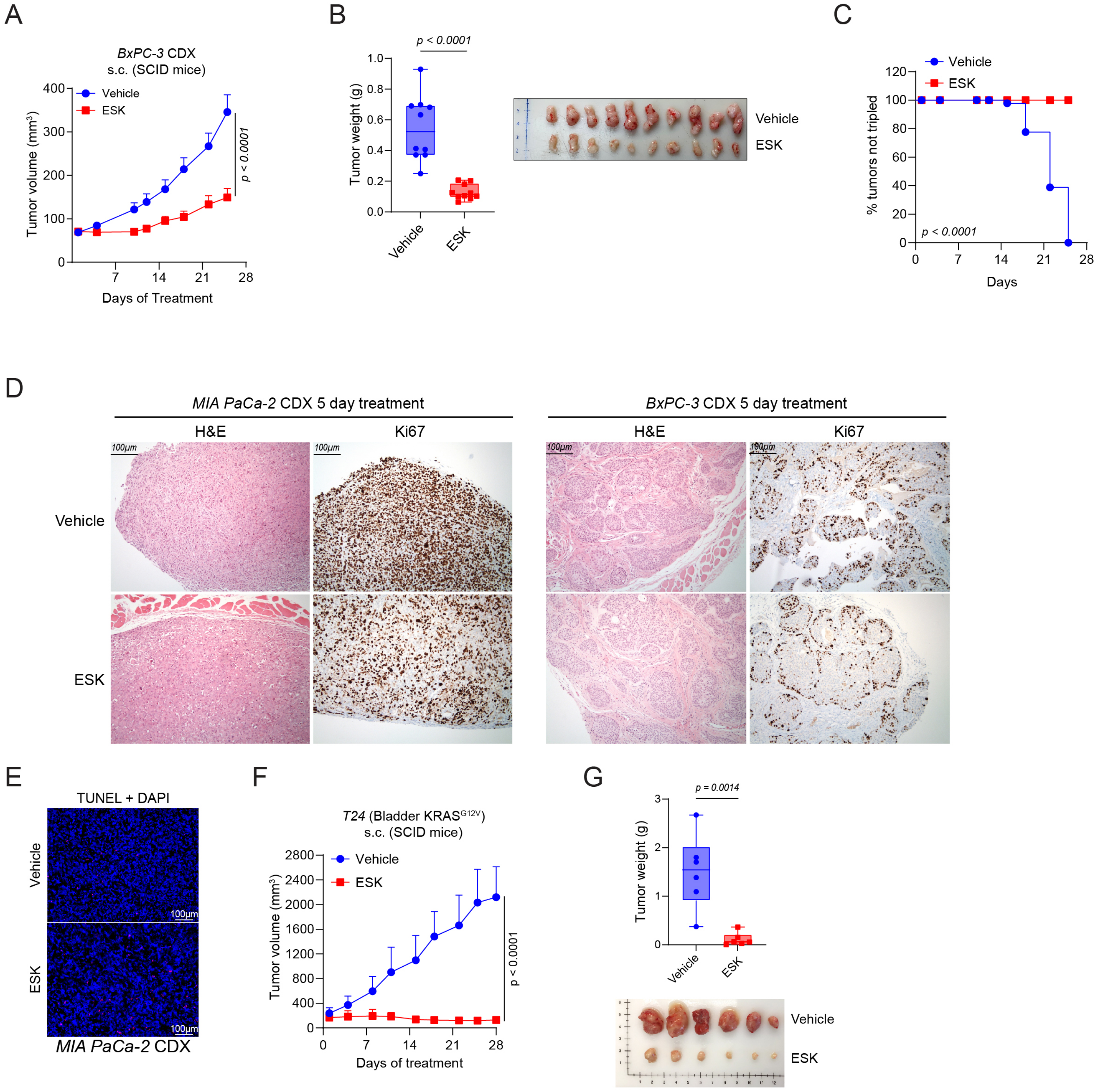

Extended Data Fig. 3

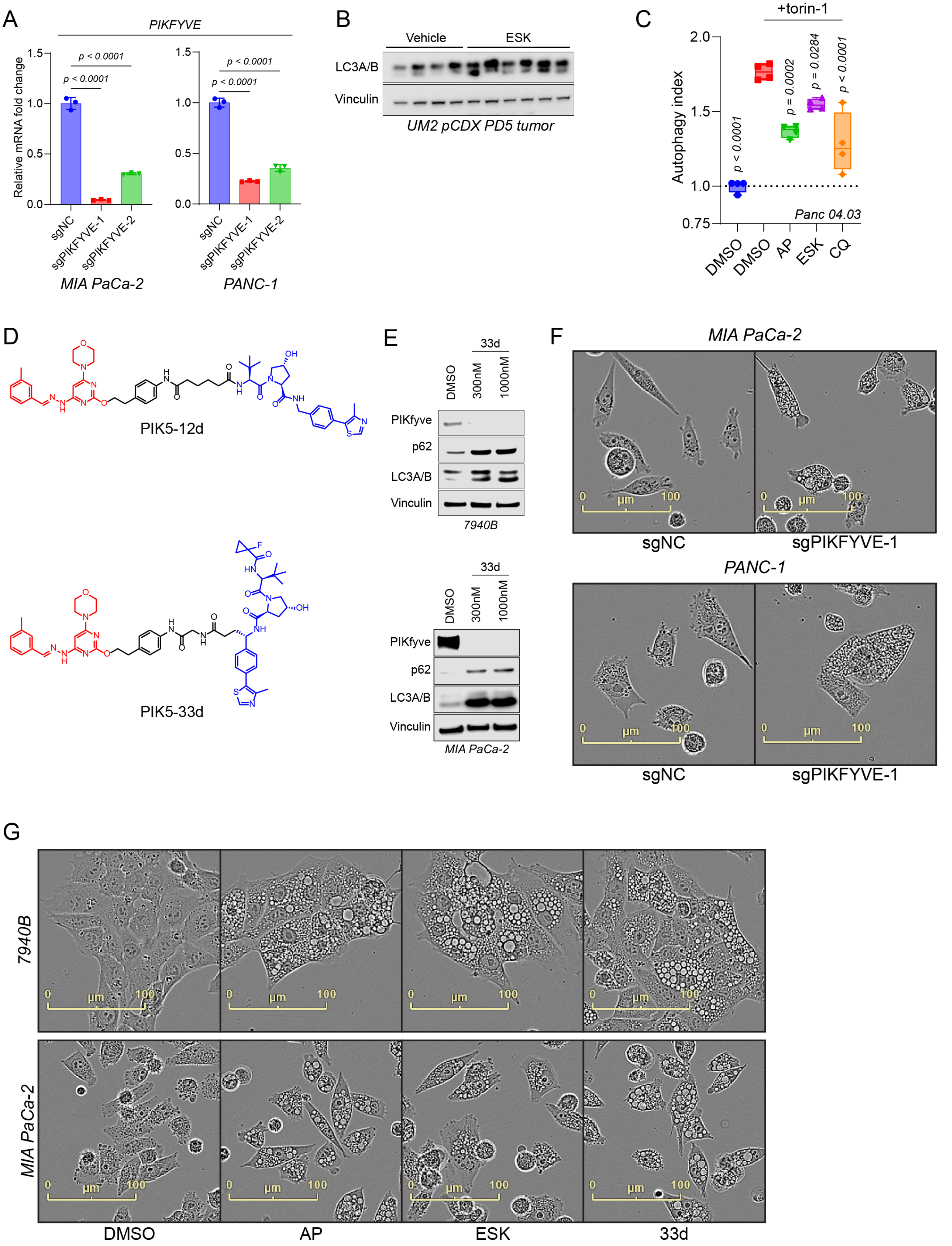

Extended Data Fig. 4

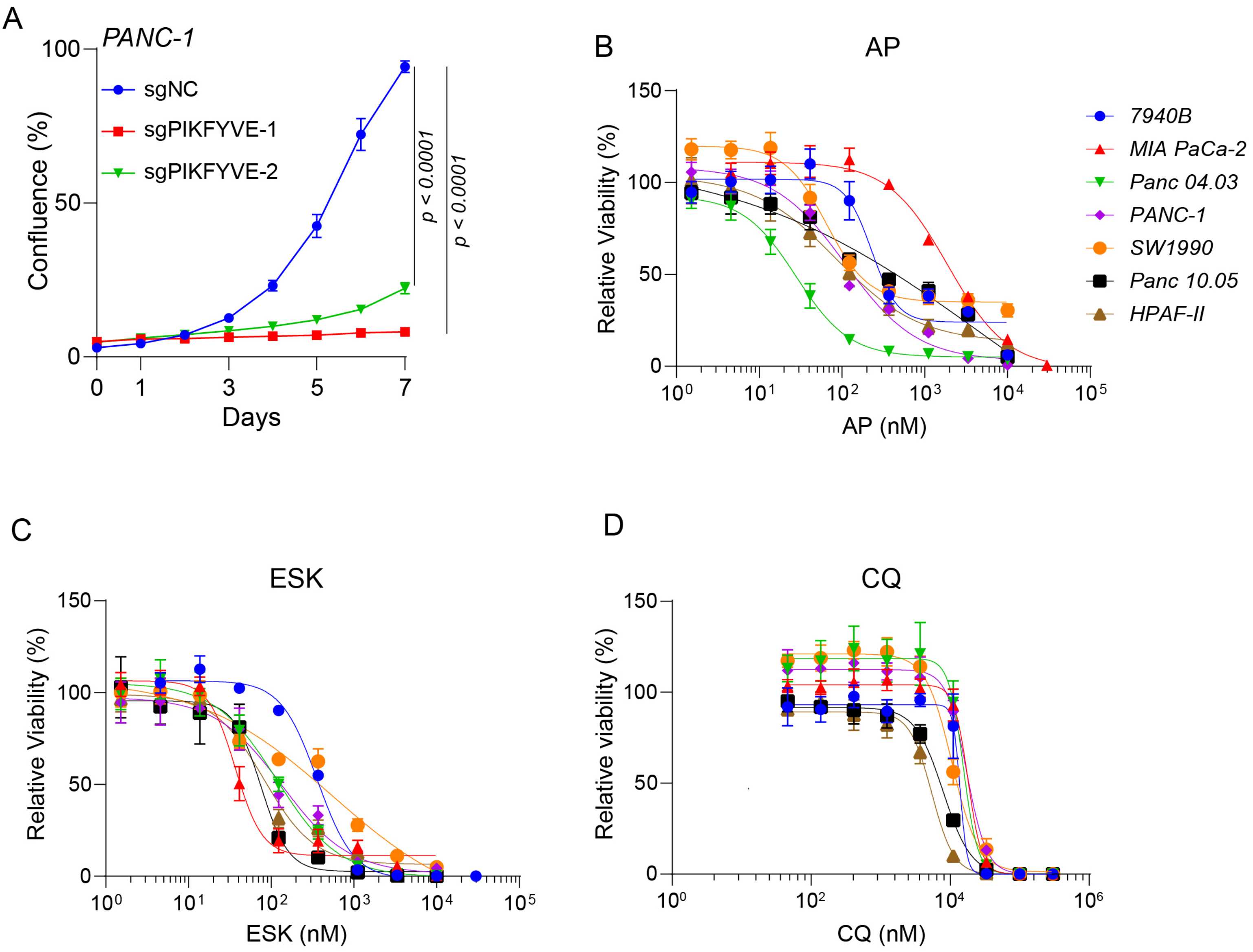

E

	AP (nM)	ESK (nM)	CQ (nM)
KPC-7940B	228.6	361.4	13893
MIA PaCa-2	1902	36.95	18019
Panc 04.03	27.97	117.8	15414
PANC-1	110.4	135.3	17306
SW 1990	65.19	554.7	10641
Panc 10.05	4960	75.22	7913
HPAF-II	85.82	81.96	5446

Extended Data Fig. 5

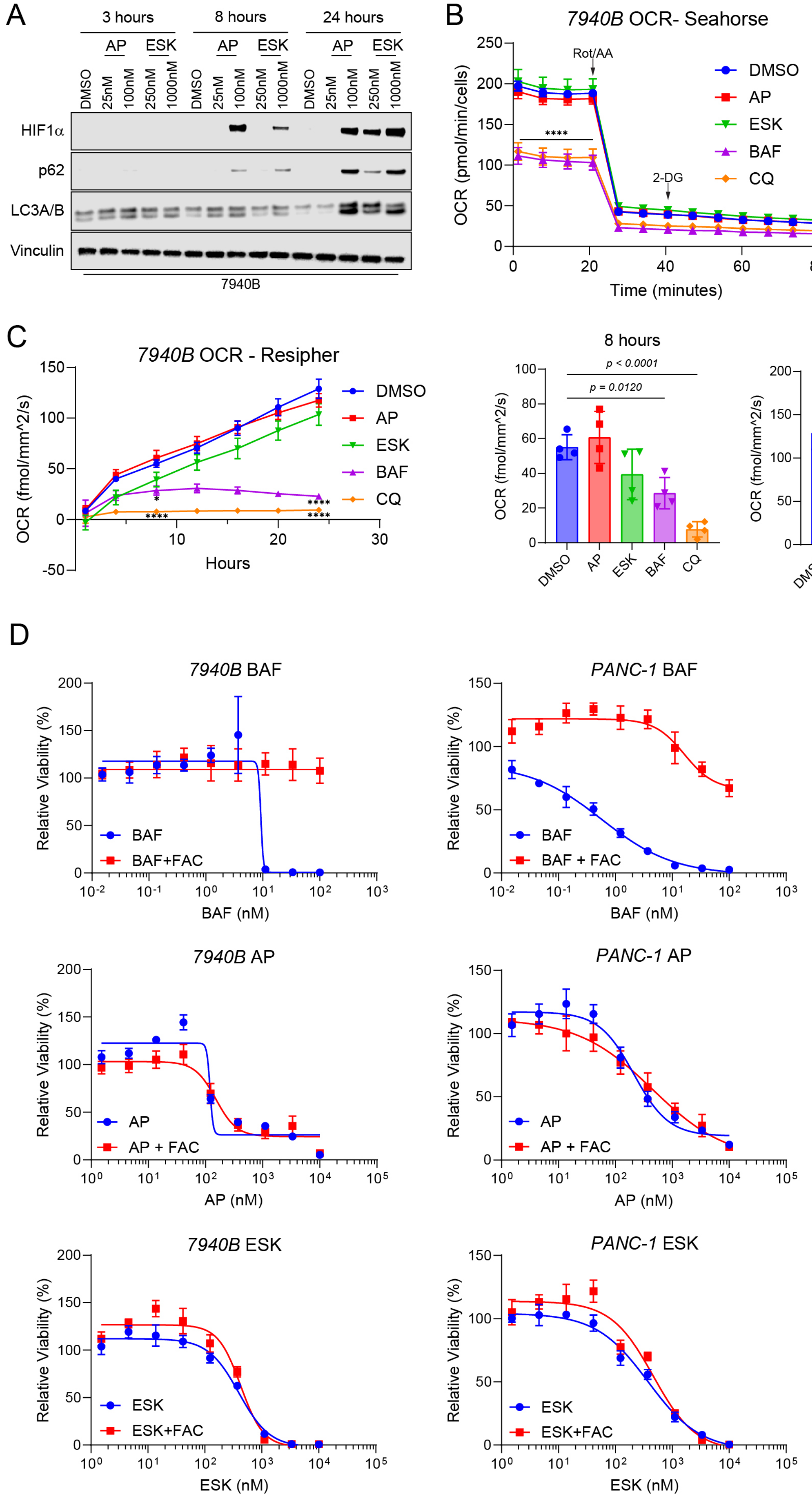

E

Half-maximal inhibitory concentration (IC_{50})

7940B	-FAC	+FAC
BAF	9.241	N/A
AP	120.1	151.8
ESK	395.7	425.9

PANC-1	-FAC	+FAC
BAF	0.5775	16.3
AP	210.7	449.8
ESK	377.4	464.3

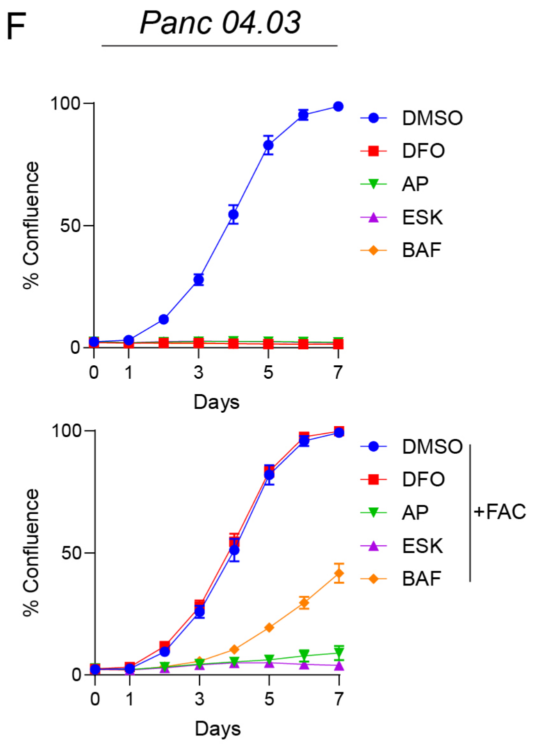

Extended Data Fig. 6

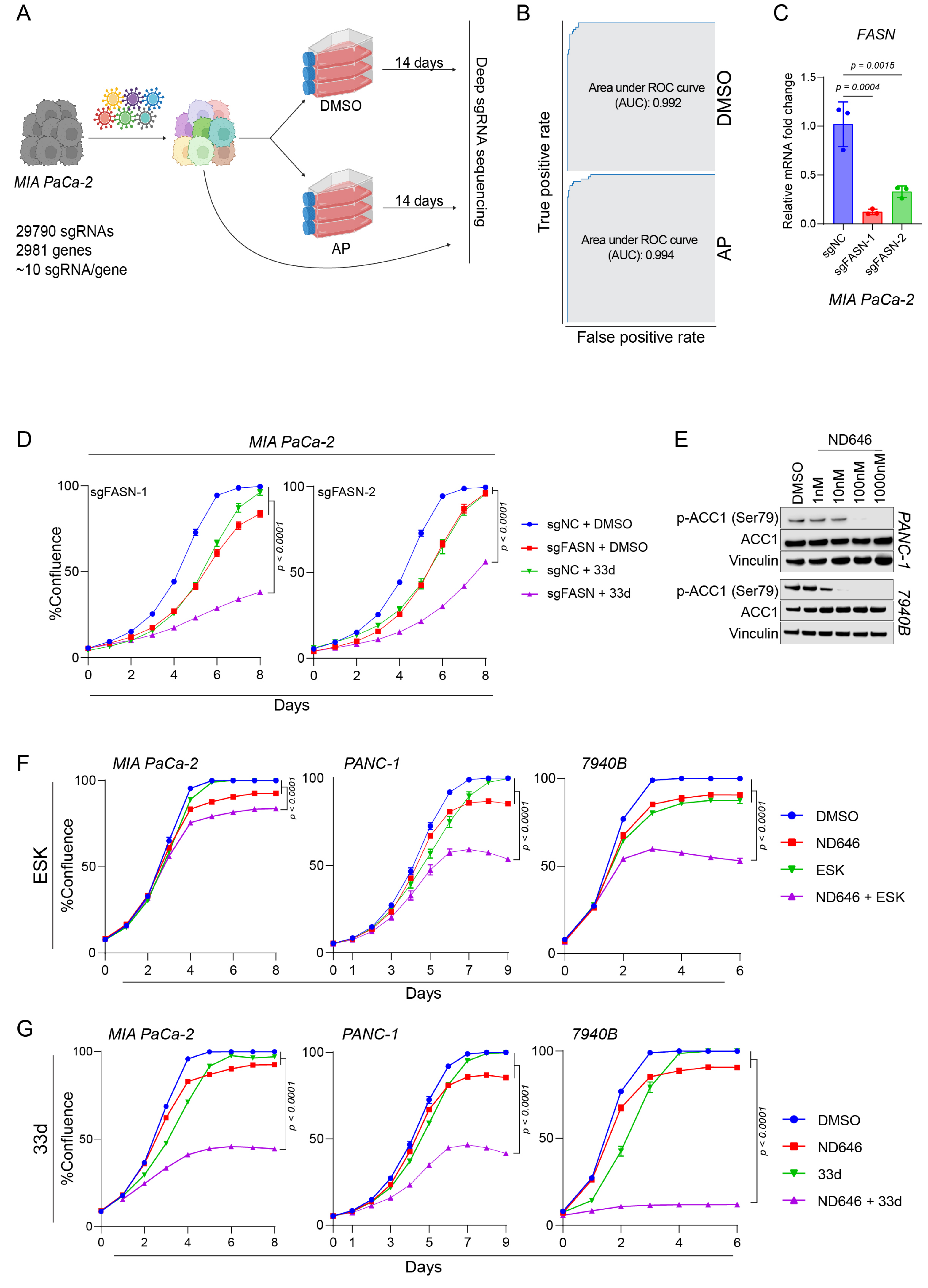

Extended Data Fig. 7

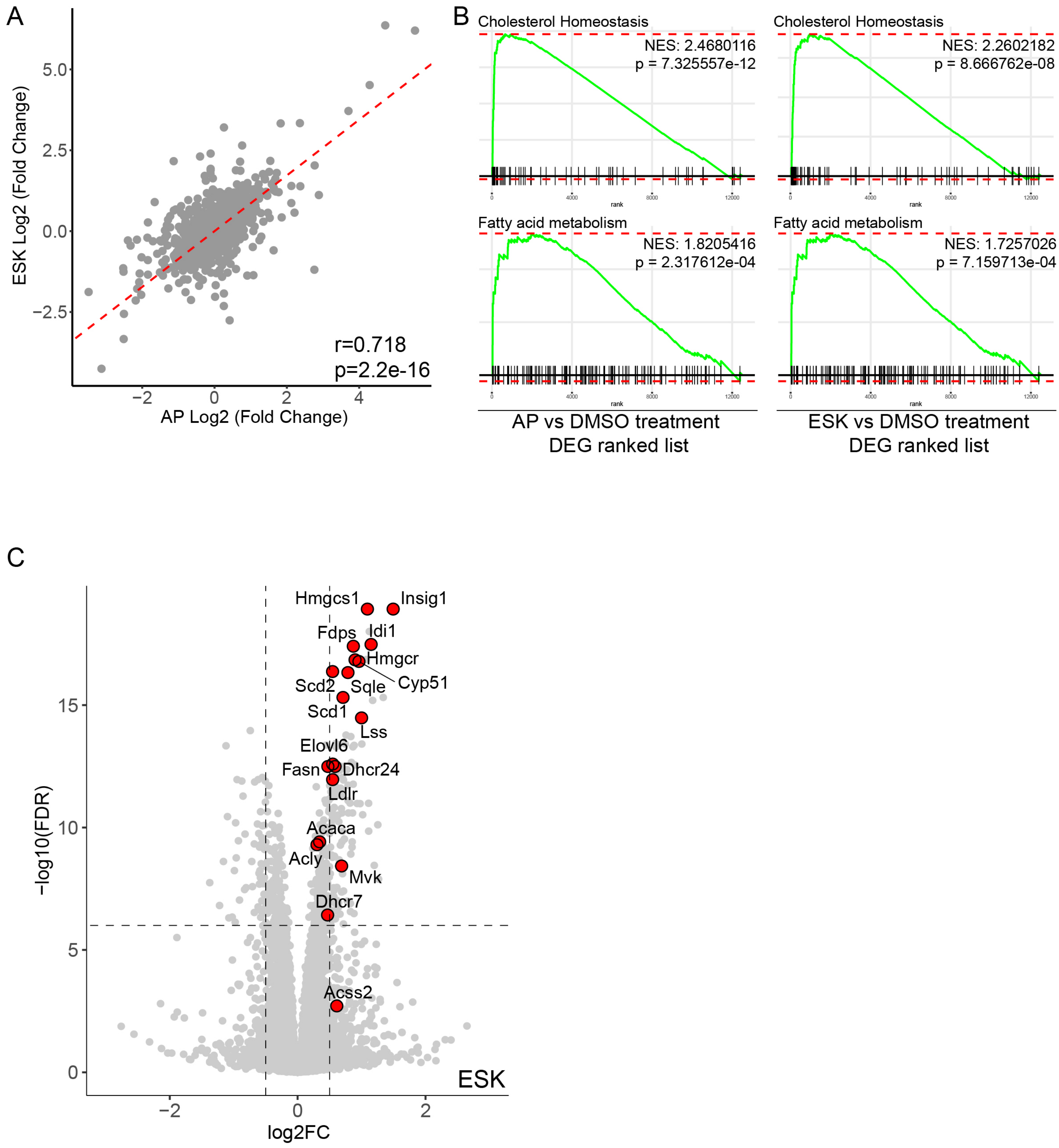

Extended Data Fig. 8

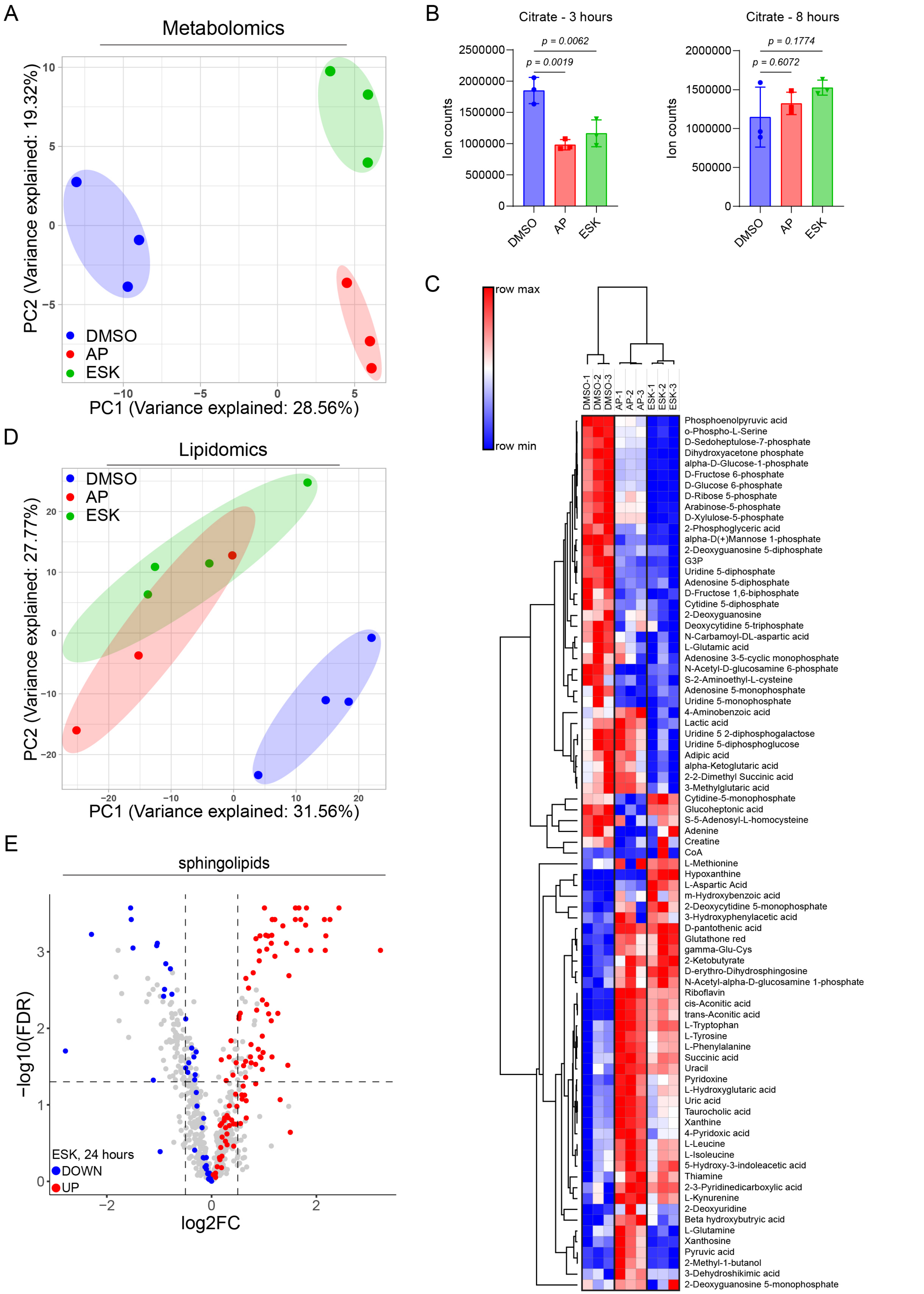

Extended Data Fig. 9

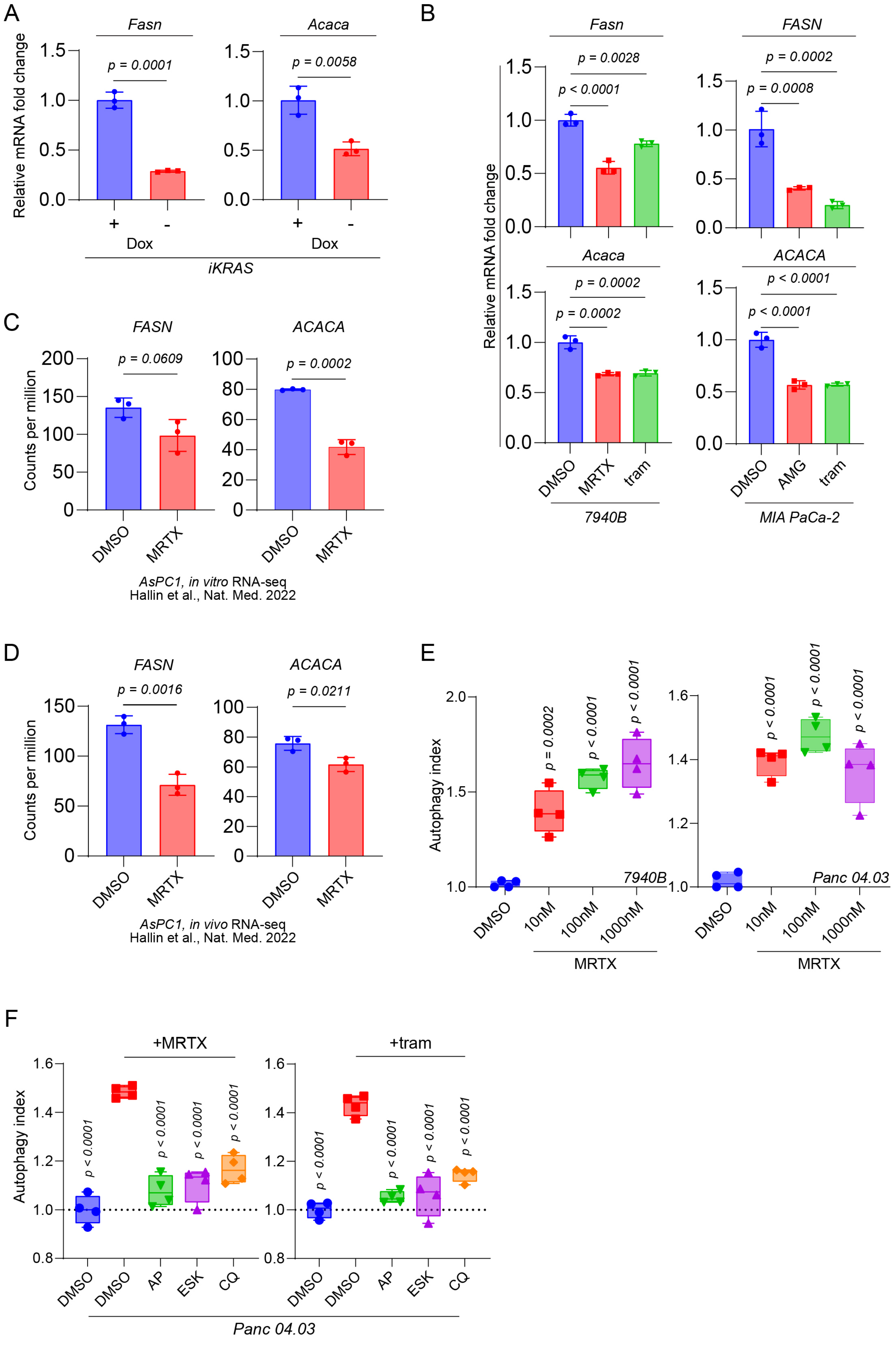

Extended Data Fig. 10

A

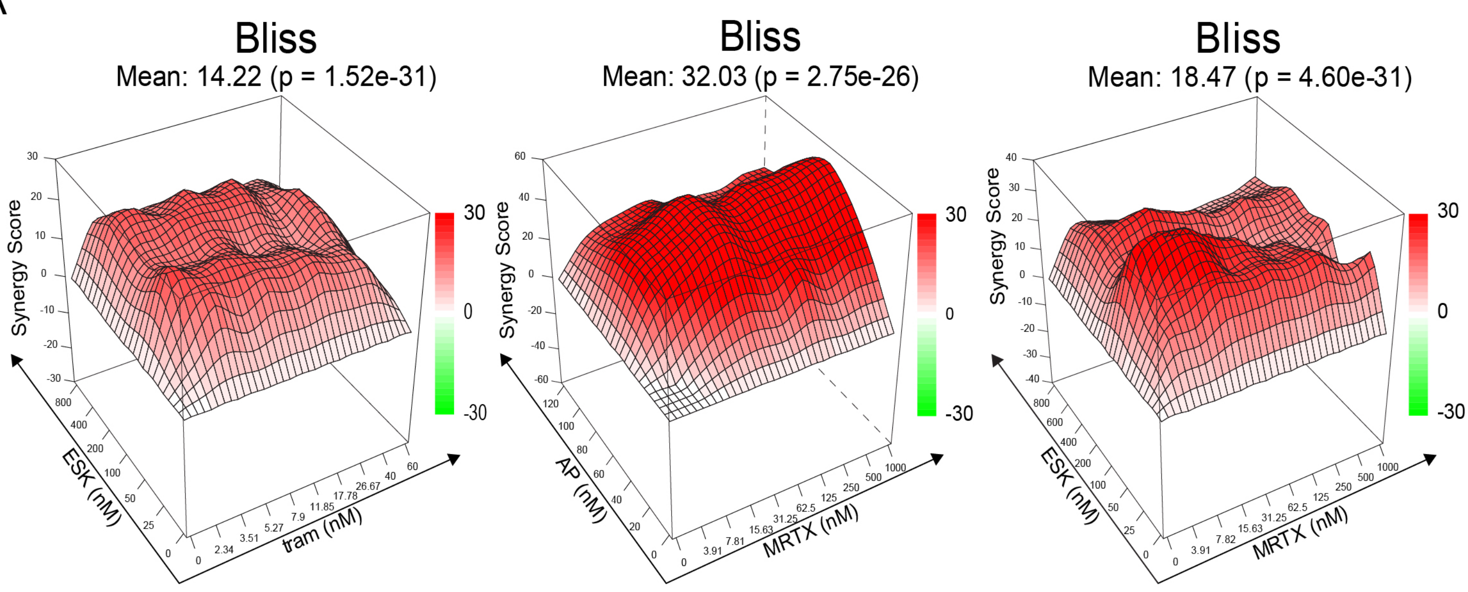

7940B

B

tram (nm) \ ESK (nM)	0	25	50	100	200	400	800
60	71	75	80	86	83	86	95
40	57	70	76	83	82	85	94
26.7	51	64	70	71	72	82	91
17.8	36	51	60	62	65	77	87
11.9	26	39	52	53	58	69	82
7.9	18	33	47	52	51	66	80
5.27	15	26	46	47	49	60	79
3.51	20	36	48	52	50	67	80
2.34	10	18	43	44	41	58	78
0	0	-1	13	21	18	34	61

MRTX (nM) \ AP (nM)	0	20	40	60	80	100	120
1000	3	54	65	83	89	90	91
500	24	53	59	78	88	88	90
250	24	47	56	74	85	85	85
125	16	49	48	65	79	82	83
62.5	11	34	41	57	70	80	78
31.3	-4	24	35	50	67	79	79
15.6	-11	14	24	37	58	70	75
7.82	-8	9	19	34	52	68	73
3.91	-7	-2	8	25	43	63	67
0	0	5	-6	3	22	46	55

MRTX (nM) \ ESK (nM)	0	25	50	100	200	400	600	800
1000	28	44	41	53	74	81	89	91
500	32	44	55	49	72	82	90	89
250	27	45	58	52	68	82	87	87
125	23	38	53	48	63	77	85	85
62.5	15	32	50	47	61	77	83	83
31.3	5	20	39	42	56	74	80	80
15.6	-6	10	39	37	55	72	82	81
7.82	-9	0	34	36	52	67	82	82
3.91	-12	-4	21	29	49	59	80	82
0	0	-8	12	9	40	47	65	70

7940B

C

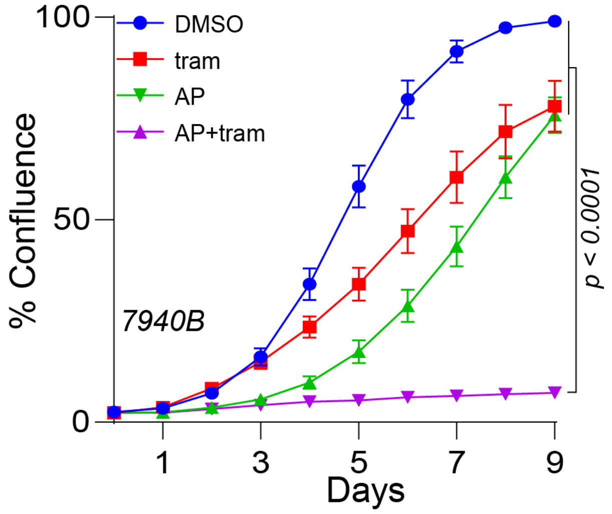

Extended Data Fig. 11

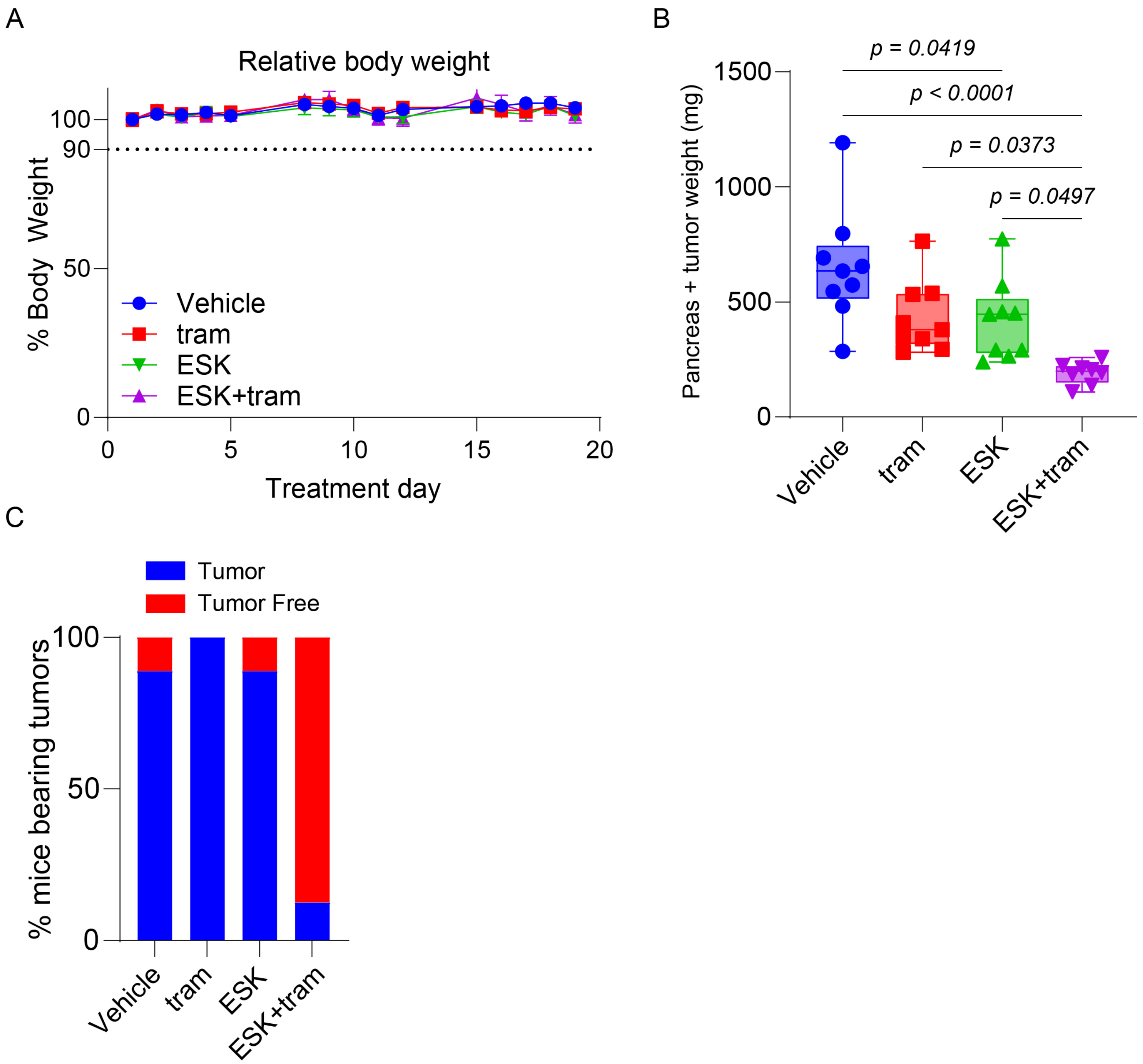

Supplement: Supplement 1 — Extended Data Fig. 1: PIKfyve is essential for progression of precursor PanIN lesions to PDAC. A. Representative image of one additional human PDAC patient sample showing H&E (left and middle) or PIKFYVE RNA-ISH (right). Scalebars are 200μm (left), 20μm (middle), 20μm (right, low magnification), and 10μm (right inset, high magnification). B. Pancreas tissue weight normalized to total body weight for Ptf1a-Cre;Pikfyve+/+ , Ptf1a-Cre;Pikfyvef/+, and Ptf1a-Cre;Pikfyvef/f mice. (One-way ANOVA with Dunnett’s) C. Representative images of H&E and insulin IHC staining from the pancreas tissue of Ptf1a-Cre;Pikfyve+/+ , Ptf1a-Cre;Pikfyvef/+, and Ptf1a-Cre;Pikfyvef/f mice. Scalebar = 50μm. D. Representative images of PIKfyve BaseScope staining from pancreas tissue of 27-week-old KC Pikfyve+/+, KC Pikfyvef/+, and KC Pikfyvef/f mice. Scalebar = 60μm for zoomed-out images; 30μm for zoomed-in images. E. Pikfyve levels as determined by BaseScope of KC Pikfyve+/+, KC Pikfyvef/+, and KC Pikfyvef/f murine pancreas tissue separated by normal and lesional areas. (One way ANOVA with Dunnett’s multiple comparisons test between the indicated groups). F. Pancreas tissue weight from 27-week-old KC Pikfyve+/+, KC Pikfyvef/+, KC Pikfyvef/f, and age-matched wild-type (WT) mice. (One-way ANOVA with Dunnett’s) G. Pancreas tissue weight normalized to total body weight (left) and raw pancreas tissue weight (right) from 40-week old KC Pikfyve+/+, KC Pikfyvef/+, and KC Pikfyvef/f, and age-matched wild-type (WT) mice. (One-way ANOVA with Dunnett’s) H. Percentage of pancreas occupied by normal tissue as determined by histological analyses of KC Pikfyve+/+, KC Pikfyvef/+, and KC Pikfyvef/f mice at 40 weeks of age. (One-way ANOVA with Dunnett’s) I. Representative images of PIKfyve BaseScope staining from pancreas tissue of 25-week-old KPC PIKfyve+/+ and KPC PIKfyvef/f mice. Scalebar = 20μm J. The age at death of mice in KPC PIKfyve+/+ and KPC PIKfyvef/f cohorts that were analyzed in Fig. 1O–P. (Unpaired [file media-1.pdf]
